# A Localized Sclerosing Osteomyelitis at the Periapex of a Vital Tooth: Report of a Misdiagnosis

**DOI:** 10.5681/joddd.2011.023

**Published:** 2011-09-05

**Authors:** Fatemeh Owlia, Mohammad-Hassan Akhavan Karbassi, Narges Mirjalili, Shokouh Taghipour Zahir, Neda Gholami, Sharare Karimi

**Affiliations:** ^1^Assistant Professor, Department of Oral Medicine, Shahid Sadoughi University of Medical Sciences, Yazd, Iran; ^2^Assistant Professor, Department of Pathology, Shahid Sadoughi University of Medical Sciences, Yazd, Iran; ^3^Post-graduate Student, Department of Oral Medicine, Shahid Sadoughi University of Medical Sciences, Yazd, Iran

**Keywords:** Chronic sclerosing osteomyelitis, chronic trauma, osteoblastoma

## Abstract

Osteomyelitis is an inflammatory infectious condition of bones, occurringeither acutely or chronically. The clinical course of the disease leads to destructive or sclerosing patterns of the involved bone. This report presents a case of chronic focal sclerosing osteomyelitis in a 19-year-old male with a history of an uncontrolled convulsive condition. The lesion was first diagnosed as an osteoblastoma. Chronic trauma or traumatic occlusion has the potential to induce osteomyelitis and should be considered a possible diagnosis in differentiating periapical radiopacities, even in relation with vital teeth.

## Introduction

Osteomyelitis is an inflammatory infectious condition of bones. It occurs either acutely or chronically, depending on the virulence of contributing pathogens, the immune system efficacy and the remodeling capacity of the involved bones. ^1^ The clinical course of the disease leads to destructive or sclerosing patterns of the involved bone. Low-virulent bacteria plus a young immunocompetent patient with a high capacity for bone remodeling is more probable to show a sclerosing form of the condition. ^2^ Osteomyelitis is rare in young people except when there is a traumatic condition. ^3^

## Case report

A 19-year-old man with a chief complaint of severe continuous pain referred to the Department of Oral Medicine, Shahid Sadoughi University of Medical Sciences. The patient felt pain in the periapical areas of maxillary right second premolar and first molar teeth (teeth #3 and #4). Pain had initiated about 20 days previously and exacerbated during the week before. It had a severe, deep, dull nature and was poorly responsive to analgesics. The patient’s medical history was non-contributory except for an uncontrolled convulsive condition with the last attack occurring three months before.

**Figure 1:**
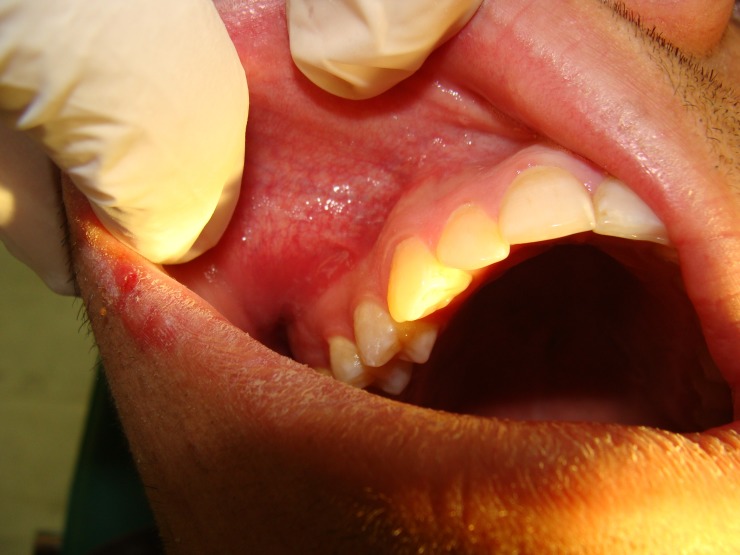
Intraoral view of the lesion that shows bony expansion on the buccal aspect of the premolar and molar region.

Extraoral examination revealed a mild asymmetry, corresponding to the painful area and a firm, non-tender, mobile submandibular lymph node, less than 1 cm in diameter. On intraoral examination, an approximately 1.5 × 2.5 cm buccal expansion of the maxillary alveolar ridge was obvious around the tooth #3. It was bony hard in palpation and covered with a smooth, intact mucosa (Figure1). All the molars and premolars of the quadrant involved were vital.

Panoramic and periapical radiographic views were ordered (Figure 2). On the panoramic view, a round radiopacity measuring 2 × 2 cm, with a relatively well-defined borders, was seen, which was fused to the roots of the first molar. In addition, a narrow radiolucent rim encircled the calcified mass, which was not present on the periapical view. It seemed that the second premolar root was pushed away mesially. With more detail, a radiating pattern of bony trabeculation could be observed on the periapical view of the lesion.


Figure 2. Panoramic (left) and periapical (right) views showing a well-defined radiopaque lesion in the apex of maxillary right first molar.

**Figure:**
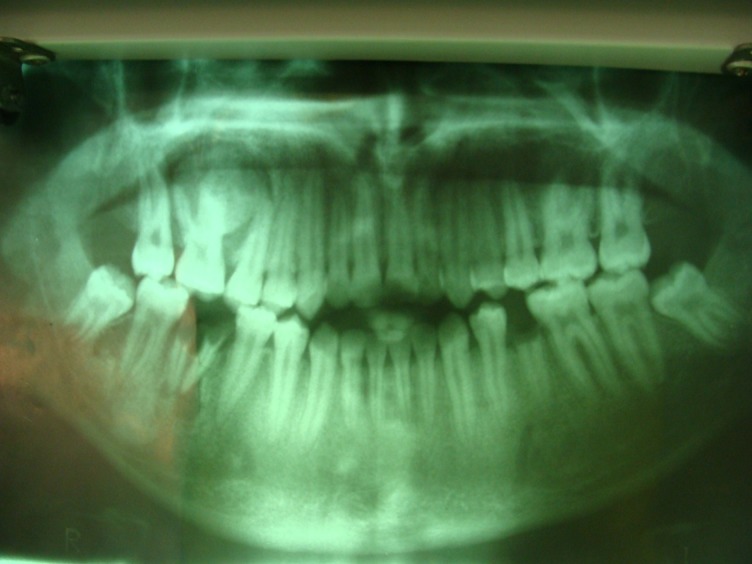

**Figure:**
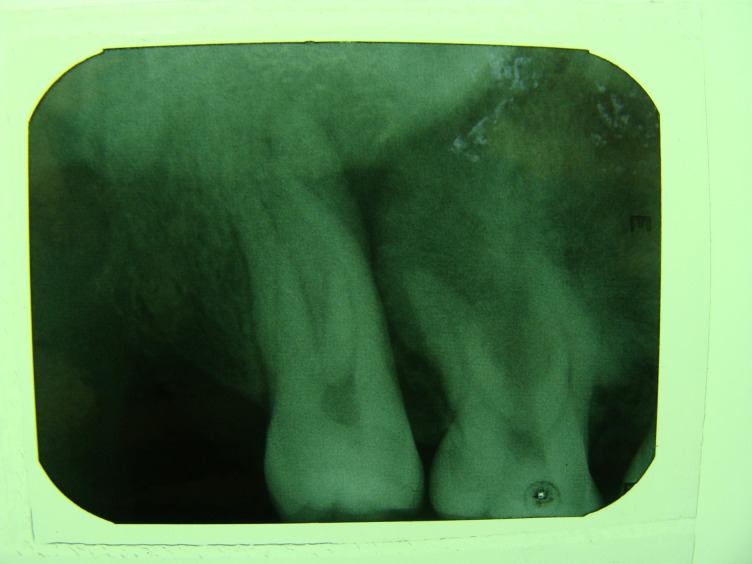

**Figure 4:**
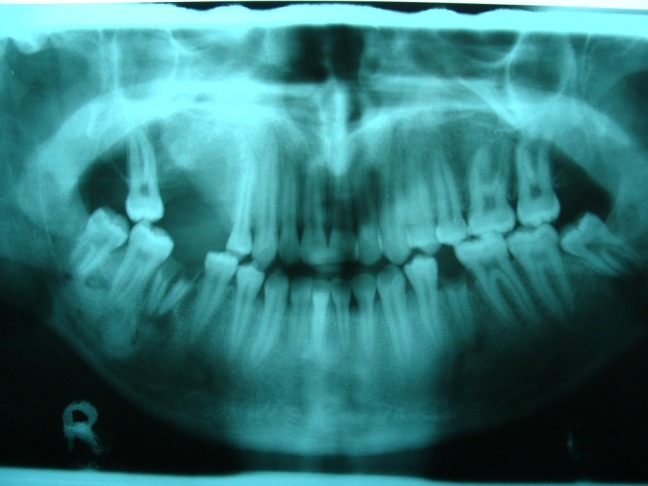
Panoramic view in one-year follow-up.

A well-defined radiopaque expansile mass in a young male adult, which did not respond well to NSAID analgesics, guided us to perform an incisional biopsy with the initial differential diagnosis of osteoblastoma. Unfortunately, the histopathological report was inconclusive but in favor of an osteoblastoma. The patient was referred to a maxillofacial surgeon for an excisional biopsy (Figure 3a). Contrary to expectations, the histopathological report documented a typical view of sclerosing osteomyelitis (Figure 3b,c). The result reported was confirmed by a second pathologist. The patient was asymptomatic in the one-year follow-up (Figure 4).


Figure 3. Enucleated lesion (a). The histological views of the lesion (b & c).

**a:**
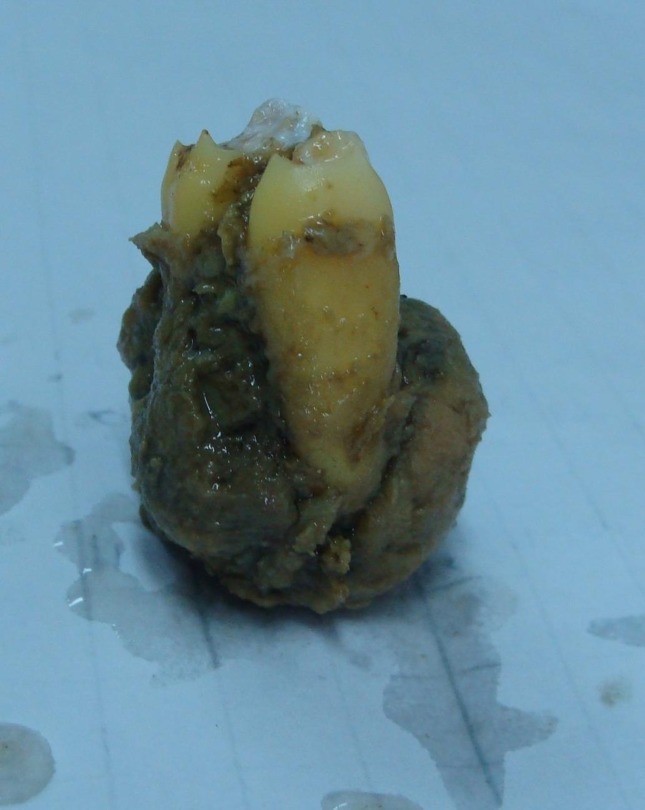

**b:**
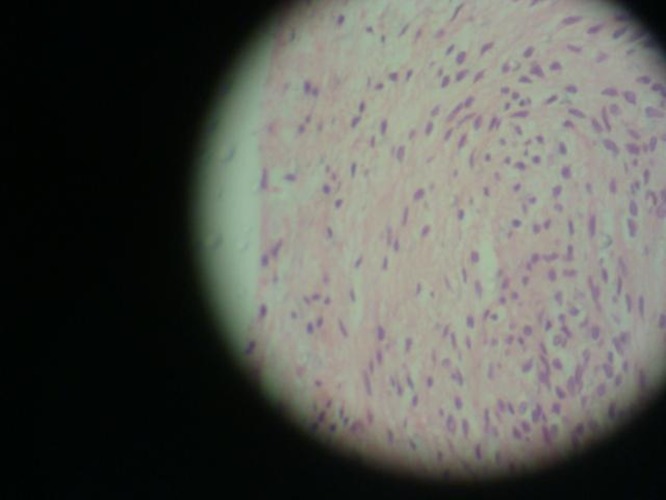

**c:**
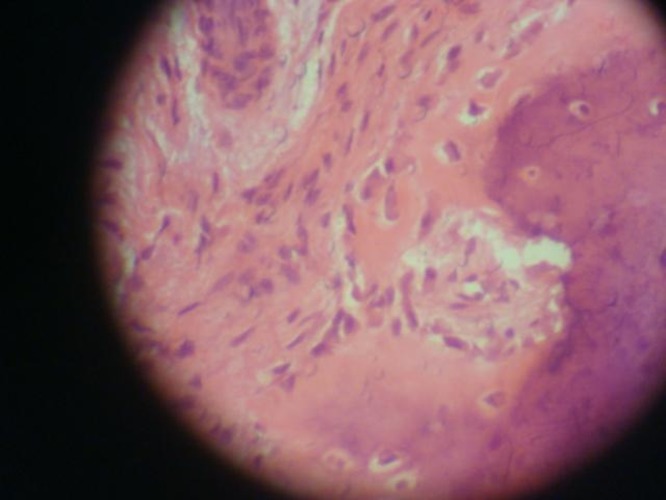

## Discussion

Approaching a pathologic condition in a systematic manner is the cornerstone of any diagnostic challenge. ^4^ Accordingly, a list of differential diagnosis for a single localized radiopaque lesion of jaw should have been arranged in this case. The so-called list included idiopathic osteoscelerosis, chronic localized sclerosing osteomyelitis, cemento-osseous dysplasia (FCOD), cementoblastoma, osteoid osteoma, osteoblastoma and osteogenic osteosarcoma.

Considering the clinical picture of the current patient, one may choose painful pathoses from the prepared list, including chronic localized sclerosing osteomyelitis, cementoblastoma, osteoid osteoma, osteoblastoma and osteogenic osteosarcoma.^2^ The least possible diagnosis was isosteogenic osteosarcoma. Although osteosarcoma occurs more frequently in males than in females, it is very rare and the remaining features of it, such as older age of incidence, involvement of the posterior mandible, poorly defined borders and invasiveness were not compatible with our patient.^1^

Cementoblastoma, a true neoplasm of cementoblasts, can present as a periapical radiopacity, which is fused to the roots of involved tooth at the very beginning. It usually forms at the periapical region of first mandibular molar and gradually expands the involved bone. A distinct radiolucent hallow almost always surrounds the central calcified mass of cementoblastoma. The opaque materials are cementoids and are not arranged in a radiating pattern.

Osteoid osteoma and osteoblastoma may exhibit completely the same features except for their response to nonsteroidal anti-inflammatory drugs (NSAIDs). Osteoblastoma has a peak incidence in the second decade of life and does not respond to NSAIDs. It originates mainly from the basal bone of posterior mandible and may affect teeth as they erupt but does not commonly initiate near the apical area of a posterior tooth.

Osteomyelitis is an infectious process with a spectrum of clinical demonstrations from a localized small sclerosing lesion to a generalized suppurative destructive one. It represents the interactions between host immunity and microbial virulence.^2^ With a fully competent immune defense, intact osseous tissue is able to restrict the infectious pathogens or kill them.^4^ Remodeling capacity of the involved bone has an important role in reactive bone formation following the inflammatory processes. ^5^These reactive calcifications may show snowflake, cotton-wool or even sun ray appearances. It is a critical rule that traumatic, infectious and benign reactive lesions are more common than malignant pathoses in the oral cavity. Osteomyelitis may share some radiographic features with primary or metastatic malignancies of the jaw. ^6^In the presented case, we would put the localized sclerosing osteomyelitis in top ranking of our differential diagnosis if we remembered that chronic traumatic conditions may induce osteomyelitis by causing microinfarctions. ^7 -9^ The patient suffered from an untreated convulsive disease. Panoramic view of his jaws signified a sclerotic thickening of cortical bone around the mandibular premolars and some hyperostotic reactions in the lower left quadrant. These findings indicate a chronic occurrence of heavy occlusal trauma in a young bone with a high remodeling capacity.

## Conclusion

Chronic trauma or traumatic occlusion has the potential to induce osteomyelitis and should be considered as a possible diagnosis in differentiating periapical radiopacities, even in relation to vital teeth.
